# Genomic and Metabolomic Polymorphism among Experimentally Selected Paromomycin-Resistant Leishmania donovani Strains

**DOI:** 10.1128/AAC.00904-19

**Published:** 2019-12-20

**Authors:** C. D. Shaw, H. Imamura, T. Downing, G. Blackburn, G. D. Westrop, J. A. Cotton, M. Berriman, M. Sanders, S. Rijal, G. H. Coombs, J. C. Dujardin, K. C. Carter

**Affiliations:** a Strathclyde Institute of Pharmacy and Biomedical Sciences, University of Strathclyde, Glasgow, United Kingdom; b Instituut voor Tropische Geneeskunde Nationalestraat, Antwerp, Belgium; c School of Biotechnology, Dublin City University, Dublin, Ireland; d Wolfson Wohl Cancer Research Centre, University of Glasgow, Bearsden, United Kingdom; e Wellcome Trust Sanger Institute, Hinxton, United Kingdom; f BP Koirala Institute of Health Sciences, Dharan, Nepal; g University of Antwerp, Antwerp, Belgium

**Keywords:** *Leishmania donovani*, paromomycin, metabolomics, lipidomics, genomics

## Abstract

Understanding the mechanism(s) underpinning drug resistance could lead to novel treatments to reverse the increased tolerance of a pathogen. In this study, paromomycin (PMM) resistance (PMM^r^) was induced in three Nepalese clinical strains of Leishmania donovani with different inherent susceptibilities to antimony (Sb) drugs by stepwise exposure of promastigotes to PMM. Exposure to PMM resulted in the production of mixed populations of parasites, even though a single cloned population was used at the start of selection. PMM 50% inhibitory concentration (IC_50_) values for PMM^r^ parasites varied between 104 and 481 μM at the promastigote stage and 32 and 195 μM at the intracellular amastigote stage. PMM resistance was associated with increased resistance to nitric oxide at the amastigote stage but not the promastigote stage (*P* < 0.05). This effect was most marked in the Sb-resistant (Sb^r^) PMM^r^ clone, in which PMM resistance was associated with a significant upregulation of glutathione compared to that in its wild type (*P* < 0.05), although there was no change in the regulation of trypanothione (detected in its oxidized form). Interestingly, PMM^r^ strains showed an increase in either the keto acid derivative of isoleucine (Sb intermediate PMM^r^) or the 2-hydroxy acids derived from arginine and tyrosine (Sb susceptible PMM^r^ and Sb^r^ PMM^r^). These results are consistent with the recent finding that the upregulation of the branched-chain amino acid aminotransferase and d-lactate dehydrogenase is linked to PMM^r^. In addition, we found that PMM^r^ is associated with a significant increase in aneuploidy during PMM selection in all the strains, which could allow the rapid selection of genetic changes that confer a survival advantage.

## Abstract (precis)

Understanding the mechanism(s) underpinning drug resistance could lead to novel treatments to reverse the increased tolerance of a pathogen. In this study, paromomycin (PMM) resistance (PMM^r^) was induced in three Nepalese clinical strains of Leishmania donovani with different inherent susceptibilities to antimony (Sb) drugs by stepwise exposure of promastigotes to PMM.

## INTRODUCTION

Visceral leishmaniasis (VL), a disease caused by infection with the protozoan parasites Leishmania donovani and L. infantum (syn., L. chagasi), is fatal if left untreated. There are 360 million people at risk of infection, and in 2017, the global incidence was estimated to be between 50,000 and 90,000 new VL cases each year, with most of them occurring in just six countries (1). Coinfection with HIV is associated with exacerbated disease and a poorer chemotherapeutic prognosis (2). Disease control is primarily focused on vector control and drug treatment of clinical cases, as there is no vaccine to protect people against infection. There are a limited number of drugs to treat VL, and treatment failure and/or drug resistance is jeopardizing their efficacy, as already illustrated in the cases of antimony (Sb) drugs (3, 4) and miltefosine (MIL) (5). With the expectation of new compounds, alternative drugs, such as amphotericin B (AMB; or its liposomal formulation, AmBisome), as well as combination therapy are being used as first-line treatment in some areas where VL is endemic. Paromomycin (PMM) is an aminoglycoside antibiotic that has been shown to have activity against *Leishmania* and that is increasingly tested in combination regimens (6). In Bangladesh, in combination with MIL or AmBisome, PMM showed efficacy similar to that of AmBisome monotherapy for the treatment of VL (7). A study has shown that combined sodium stibogluconate (SSG) and PMM treatment in Sudan, Kenya, Uganda, and Ethiopia was an effective first-line option for VL chemotherapy (8). A retrospective study on the treatment of post-kala azar dermal leishmaniasis (PKDL), a complication of VL, showed that combination therapy with PMM was more effective than monotherapy with SSG in East Africa (9).

Experimental work showed that the selection of PMM resistance (PMM^r^) in L. donovani was rather easy and fast (10, 11). Given the increased usage of PMM in clinical practice, it is thus important to understand the molecular mechanisms underlying PMM resistance in *Leishmania.* The drug is known to downregulate protein synthesis, and it has been shown to affect multiple protein targets in L. donovani (12). The cytoplasmic target of PMM is the decoding A site of the small subunit (SSU) of ribosomes, where it increases misreading and translation inhibition. Steric hindrance caused by a 1408A-to-G mutation at the gene region encoding the 18S rRNA helix h44, which forms part of the A site or the rRNA, was thought to be responsible for the poor binding of PMM to eukaryotic cytosolic ribosomes. Mutations at positions 1408 and 1491 are known to be important in determining sensitivity to PMM (13). A study using a high-throughput cosmid sequencing (Cos-Seq) approach identified genetic changes on 5 chromosomes that conferred PMM resistance in L. infantum. However, the product of these gene sequences was unknown and Cos-Seq could not identify loss-of-function targets (14). Previous work showed that the molecular mechanisms of drug resistance may vary with the genetic background of the parasite (15). In the present study, we thus selected for PMM^r^ in three L. donovani clinical strains from Nepal with different susceptibilities to Sb drugs and different genetic backgrounds. The wild-type (WT) strains were previously phenotyped as Sb sensitive (Sb^s^), Sb intermediate (Sb^i^), or Sb resistant (Sb^r^), respectively (16). We characterized the effect of PMM^r^ on the metabolomes of the strains as well as their infectivity and susceptibility to antimicrobial products produced by macrophages. We sequenced the whole genomes of the Sb^s^ and Sb^r^ strains during induction of PMM^r^. We show polymorphic molecular changes among the different PMM^r^ strains.

## RESULTS

### PMM susceptibility (PMM^s^) upon adaptation of Sb^s^, Sb^i^, and Sb^r^ lines to PMM.

The Sb^s^ WT parent strain used here (strain BPK280/0cl4) was previously found to be intrinsically less susceptible to PMM than the other two L. donovani strains used in this study (Table 1). However, this strain was submitted to the same PMM resistance selection protocol to determine if PMM selection resulted in increased PMM resistance, as studies have shown that drug-resistant parasites can lose their drug resistance if drug pressure is removed (17). After adaptation of the parasites to grow in 97 μM PMM, promastigotes of the induced Sb^s^ PMM^r^ parent line had a lower 50% inhibitory concentration (IC_50_) value than the WT, but cloned lines from this parent had either an IC_50_ lower than that for the original WT or an IC_50_ value similar to that of the original WT after PMM selection (Table 1; data for one cloned line are shown). This may indicate that there is an upper limit of the ability of this strain to tolerate PMM exposure and that selection resulted in the expansion of different subclones from the original WT. At the amastigote stage, the Sb^s^ PMM^r^ parent and cloned line had a similar or lower susceptibility to PMM (Table 1). In contrast, selection using PMM resulted in increased resistance to PMM at both the promastigote and the amastigote stages for the Sb^i^ strain (BPK087/0cl11) and Sb^r^ strain (BPK275/0cl18). The PMM^r^ cloned lines from the PMM^r^ Sb^i^ or PMM^r^ Sb^r^ parent had similar susceptibilities to PMM (Table 1).

**TABLE 1:** Relative susceptibility (IC_50_) of different L. donovani strains to PMM

Strain phenotype	Isolate	Mean ± SD PMM IC_50_ (μM)^*a*^	
		Promastigote	Amastigote
Sb^s^	WT	354 ± 4	166 ± 11
	PMM^r^ parent	116 ± 29	149 ± 20
	PMM^r^ clone 8	305 ± 27	155 ± 26
Sb^i^	WT	56 ± 22	56 ± 18
	PMM^r^ parent	455 ± 6*	156 ± 20*
	PMM^r^ clone 8	481 ± 84*	195 ±12*
Sb^r^	WT	65 ± 2	67 ± 1
	PMM^r^ parent	455 ± 6*	165 ± 20*
	PMM^r^ clone 9	455 ± 6*	165 ± 20*

a The data show the mean IC_50_ from a minimum three separate experiments. *, *P* < 0.05 compared to the corresponding WT.

### Metabolomic profile of PMM^r^ parasites.

**FIG 1:**
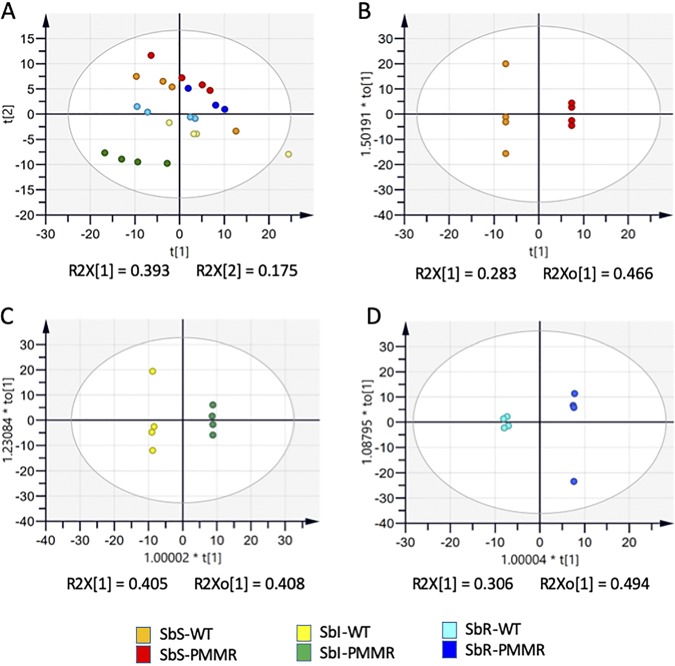
Multivariate analysis of data from LC-MS was performed using the SIMCA program. There are 214 features (metabolite peak intensity values) per sample. Plots show the scores resulting from modeling of the data matrix: PCA-X analysis of all samples (A), OPLS-DA of Sb^s^ strains (B), OPLS-DA of Sb^i^ strains (C), and OPLS-DA of Sb^r^ strains (D). Ellipses represent 95% tolerance limits (Hotelling's T-squared distribution [T^2^]) (35).

**FIG 2:**
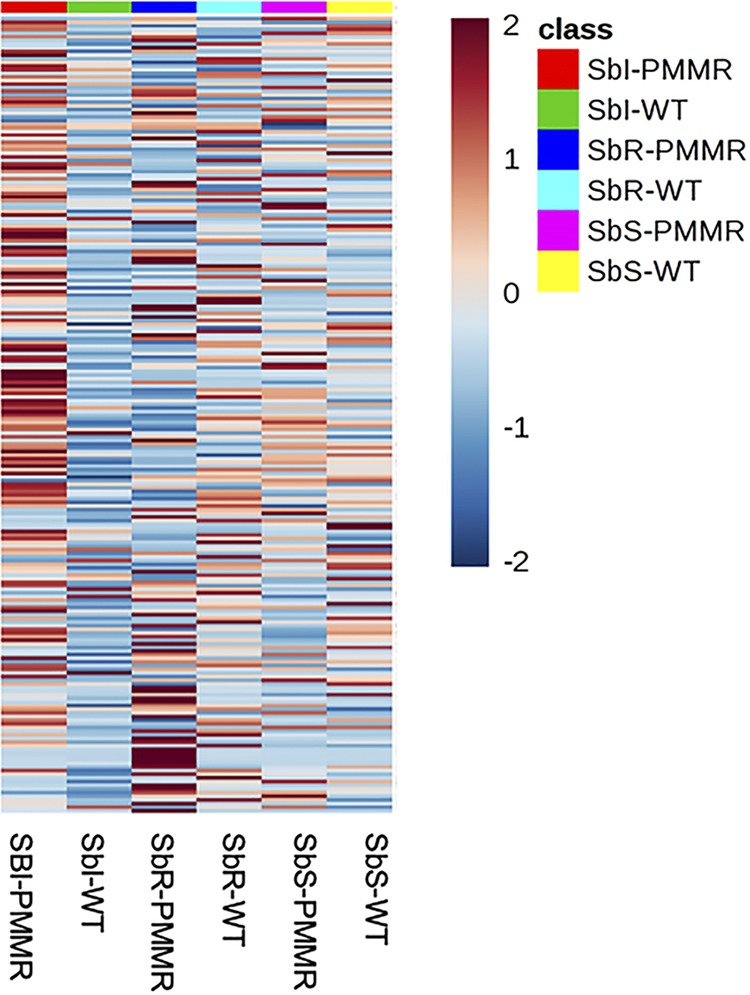
Effect of PMM selection on the metabolic profile of PMM^r^ L. donovani promastigotes compared to that of the WT. Heat maps of metabolite levels (mean peak intensity) were generated using Metaboanalyst (v4.0) software. The map shows the mean metabolite levels for the different L. donovani strains (columns) based on mean peak intensity values from LC-MS (rows). Deep red represents the highest level, and deep blue represents the lowest level, with white representing equal levels. Each group is identified by a colored bar at the top of each column and by the name of the strain at the bottom. These data are representative of those from three separate experiments.

**FIG 3:**
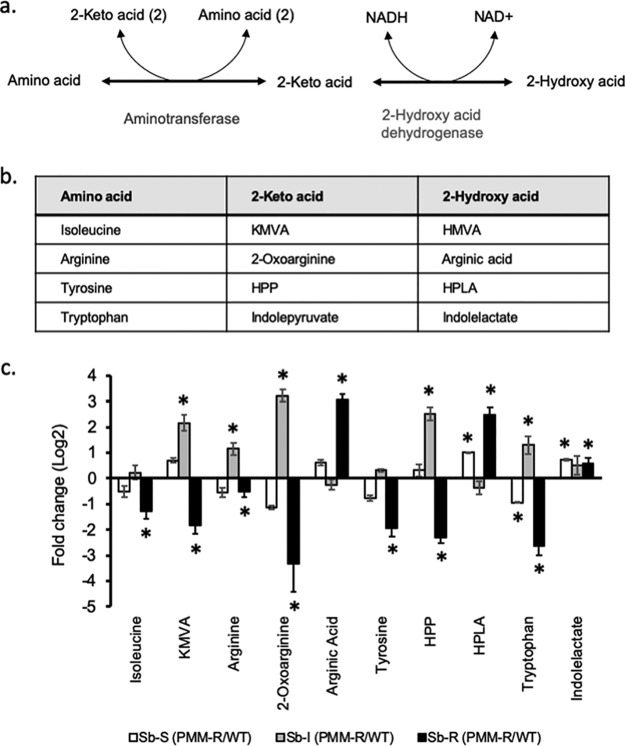
Hydroxy acid production in the different L. donovani strains. (a) General pathway for production of hydroxy acid derivatives of amino acids in *Leishmania*. (b) 2-Keto acids and 2-hydroxy acids produced from isoleucine, arginine, tyrosine, and tryptophan. (c) Differences in hydroxy acid levels in wild-type and PMM-resistant strains (log_2_ fold change in metabolite peak intensity). *, *P* ≤ 0.05. Abbreviations: KMVA, 2-keto-3-methylvaleric acid; HMVA, 2-hydroxy-3-methylvaleric acid; HPP, hydroxyphenylpyruvate; HPLA, hydroxyphenyllactate.

**FIG 4:**
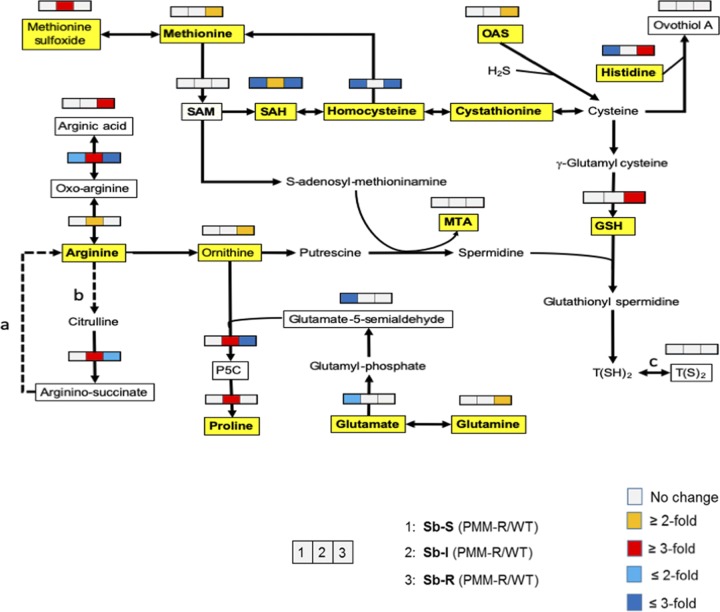
Arginine and methionine metabolism in different strains of L. donovani. A pathway map for arginine, proline, methionine, and cysteine metabolism is shown. The map also includes glutathione and trypanothione biosynthesis. Boxed metabolites were detected in the metabolic profile of the L. donovani strains by LC-MS. Yellow shading indicates that metabolite identities were confirmed by retention times matching those obtained with authentic standards; unshaded metabolites were putatively identified by accurate mass only. Unboxed metabolites were not detected by LC-MS but are presumed to be present. Abbreviations: SAM, *S*-adenosylmethionine; SAH, S-adenosylhomocysteine; MTA, 5-methylthioadenosine; OAS, *O*-acetylserine; GSH, glutathione; GSSG, oxidized glutathione; T(SH)2, reduced trypanothione; T(S)2, oxidized trypanothione; P5C, pyrroline-5-carboxylate. Filled arrows represent enzyme reactions known to occur in *Leishmania* spp. Dotted arrows show reactions that have not been confirmed. a, the urea cycle is not thought to be complete in *Leishmania*; b, nitric oxide synthase activity was detected in L. amazonensis, but the gene has not been identified in any *Leishmania* species; c, the oxidation of thiols may occur *in situ* during cell extract formation and LC-MS.

Metabolites of the three L. donovani PMM^r^ clones, i.e., Sb^s^ PPM^r^ clone 8, Sb^i^ PMM^r^ clone 8, and Sb^r^ PMM^r^ clone 9, and their corresponding WTs were extracted on the same day of culture from parasites that grew at the same rate and analyzed to identify the metabolomic changes associated with PMM resistance. The same 212 metabolites were putatively identified in all six lines (see Excel Files 1 and 1a in the supplemental material posted at https://doi.org/10.15129/88f47c26-ff93-4a7c-8c17-d323aa24052e). Principal-component analysis (PCA) showed that replicates of each of the L. donovani strains clustered into separate groups (Fig. 1A). Clear differences were obvious when each WT was compared to its corresponding PMM^r^ derivative using orthogonal projections to latent structures discriminant analysis (OPLS-DA) (Fig. 1B to D), indicating that clear metabolic differences occurred on adaptation to 97 μM PMM. Overall, there were more differences in the Sb^i^ PMM^r^ and Sb^r^ PMM^r^ strains than in the Sb^s^ PMM^r^ strain (see Tables S1 and S2 posted at the website indicated above). Figure 2 shows a heat map of the relative expression of the metabolites shown in Table S1, to highlight the effect of the induction of PMM^r^ on the metabolomic profiles of the parasites. Metabolic changes occurred in a number of biochemical pathways, i.e., amino acid metabolism, glutathione metabolism, carbohydrate metabolism, glycolysis, the tricarboxylic acid (TCA) cycle, and nucleotide metabolism. It was possible to relate some changes to specific biochemical pathways within pairs of strains. For example, *Leishmania* species produce hydroxy acid derivatives of aromatic amino acids and arginine (18). This process requires the production of a 2-keto acid by transamination of the amino acid, followed by reduction of the keto acid to form the corresponding 2-hydroxy acid (Fig. 3a), and specific products for arginine, tyrosine, and tryptophan were detected in the metabolic profile (Fig. 3b and c). Significant differences in arginine metabolites were observed, with a large decrease in 2-oxoarginine levels and a corresponding increase in arginic acid levels being seen for the Sb^r^ PMM^r^ clone compared to its WT (Fig. 3c). The same trends were found for the Sb^s^ PMM^r^ clone, although the changes were smaller and generally below the 2-fold threshold. In contrast, the Sb^i^ PMM^r^ clone had significantly higher levels of the keto acid derivatives of arginine and tyrosine (2-oxoarginine and hydroxyphenyl pyruvate [HPA], respectively) but no significant changes in the corresponding 2-hydroxy acids (arginic acid and hydroxyphenyllactate [HPLA]). The changes in 2-hydroxy acids indicate an increased amount of 2-hydroxy acid dehydrogenase, although the metabolic enzymes responsible for this activity have not been identified in *Leishmania*. This activity could be important for NAD/NADH recycling to maintain the redox balance within the promastigote, so the change could be an indirect effect of PMM^r^. The levels of tryptophan varied between the strains, but there was no corresponding difference in the levels of its hydroxy acid derivative, indolelactate. Arginine is involved in a number of metabolic pathways (Fig. 4) and could affect proline levels. Proline has been shown to protect against oxidative stress, and its upregulation has been associated with drug resistance (18). Proline levels were upregulated in the Sb^i^ PMM^r^ clone compared to those in its WT but not the other two PMM^r^ clones. Therefore, in order to determine if proline could mediate PMM resistance in the Sb^s^ and Sb^i^ strains, the WT strains and their corresponding PMM^r^ clones were exposed to PMM at different concentrations in medium supplemented with proline. Addition of proline to the medium had no significant effect on parasite growth or the response to PMM treatment, giving further evidence that proline is not involved in PMM^r^. Trypanothione, the main intracellular thiol and reducing agent in trypanosomatids, was detected (in its oxidized form), but there were no differences observed between WT strains and their PMM^r^ derivatives (Fig. 4). It should be noted that thiols can be oxidized *in situ* during liquid chromatography-mass spectrometry (LC-MS), and the levels observed are indicative of the total amount in the extract rather than their oxidation state *in vivo*. Other compounds can also protect against oxidative stress, and in the Sb^r^ PMM^r^ clone, there was a significant upregulation in the levels of glutathione (*P* < 0.05). However similar metabolic changes were not present in the other two PMM^r^ clones (Fig. 4). PMM^r^ had little effect on the glucose metabolism of the parasites, as the levels of the majority of the metabolites in the TCA cycles were unaffected compared to those in the WT strains (Fig. S1). This could indicate that PMM^r^ does not require active transport mechanisms that require energy.

### Lipidomic profile of PMM-resistant parasites.

The regulation of none of the lipids identified in lipidome studies was significantly altered after PMM^r^ selection for the Sb^i^ PMM^r^ strain compared to that in its WT (Table S2). In contrast, 7 lipids were significantly downregulated and 1 lipid (sphingenine) was upregulated in the Sb^r^ PMM^r^ strain compared to their regulation in its WT. Of these 7, 4 lipids were small, unsaturated phosphatidylethanolamines (PEs; two diacyl PEs, one alky-acyl PE, and one alkenyl-acyl PE). The other three were a ceramide, a phosphatidylinositol, and a lysophosphatidylethanolamine (Table S2). In the Sb^s^ PMM^r^ strain, there was a significant upregulation of 4 alkyl-acyl phosphatidylinositols. There was also a significant reduction of 4 other lipids that are not structurally related.

### PMM^r^ does not affect infectivity but influences macrophage responses.

In previous studies of MIL-resistant (MIL^r^) parasites derived from the same WT parents, we found that MIL^r^ was associated with a reduction in macrophage infectivity (19). Therefore, studies were carried out to determine if PMM resistance affected parasite fitness by assessing their ability to infect macrophages and their susceptibility to the antimicrobial products produced by macrophages. PMM^r^ was not associated with a reduction in infectivity for macrophages, as parasite levels were similar in macrophages infected with WT or PMM^r^ parasites (data not shown). Sb^s^ PMM^r^ parasites had a significantly greater resistance to gamma interferon (IFN-γ)- and lipopolysaccharide (LPS)-induced killing than the WT (*P* < 0.05), but only if cells were treated with the lower doses of IFN-γ and LPS (i.e., 50 units IFN-γ and 50 ng LPS/ml versus 100 units IFN-γ and 100 ng LPS/ml; data not shown). The Sb^i^ and Sb^r^ PMM^r^ parasites did not demonstrate a consistent difference in susceptibility to IFN-γ- and LPS-induced killing from their corresponding WT at either dose (data not shown). Treatment with *S*-nitroso-*N*-acetyl-d,l-penicillamine (SNAP), a nitric oxide (NO) inducer, caused a dose-dependent reduction in parasite survival, and the effect was not related to any difference in NO production, as similar amounts of nitrite were present in the supernatants of uninfected and infected macrophages treated with SNAP (data not shown). The Sb^s^ WT and Sb^s^ PMM^r^ promastigotes had similar tolerances to SNAP-induced parasite killing, whereas the Sb^i^ PMM^r^ and Sb^r^ PMM^r^ clones were less resistant to NO exposure than their respective WT strains (Table 2). At the intracellular amastigote stage, all independently cloned Sb^s^ promastigotes tested except one, Sb^s^ PMM^r^ clone 6, were significantly more resistant to SNAP treatment than their WT counterparts (*P* < 0.05; only data for clone 8 are shown in Table 2). The anomaly for Sb^s^ PMM^r^ clone 6 may be because this clone had the same PMM susceptibility as the Sb^s^ WT (data not shown). The Sb^r^ WT exhibited the highest resistance to SNAP treatment at the promastigote stage of the three WT strains, with an IC_50_ value that was 9 times higher than that for the other two WT strains. This may reflect the inherent resistance of this strain to Sb, as Sb^r^ is related to enhanced resistance to reactive nitrogen species (20). This may explain why the increase in SNAP resistance for Sb^r^ PMM^r^ clone 9 at the amastigote stage was only 1.3 times higher than that for the Sb^r^ WT, whereas the other PMM^r^ parasites expressed a much higher differential resistance to SNAP treatment (resistance relative to that of the WT was 2.1 to 2.6 times higher for the Sb^s^ PMM^r^ strain than for the Sb^s^ WT and 25.3 times higher for the Sb^i^ PMM^r^ strain than for the Sb^i^ WT). 3-Morpholinosydnonimine (SIN-1) treatment, which induces both NO and superoxide production in macrophages, did not give a clear differentiation in the fitness of the WT and the PMM^r^ parasites, based on the IC_50_ value for SIN-1 exposure (Table 2). The Sb^s^ PMM^r^ and Sb^r^ PMM^r^ parasites had susceptibility to SIN-1 similar to that of their corresponding WT strains, whereas Sb^i^ PMM^r^ clone 8 promastigotes were significantly more susceptible to SIN-1 treatment than the Sb^i^ WT (*P* < 0.05). There was a difference in SIN-1 susceptibility between the WT strains, with the Sb^i^ WT being the most resistant and the Sb^r^ WT being the least resistant (Table 1). Overall, the most striking phenotype associated with PMM^r^ was increased resistance to NO, measured at the IC_50_ value for SNAP exposure in the amastigote stage of the parasite. The Sb background of the strains did have an influence, with the Sb^r^ PMM^r^ strain exhibiting greater resistance to NO.

**TABLE 2:** Susceptibility (IC_50_) of different L. donovani strains to SIN or SNAP treatment

Strain	Mean ± SD IC_50_ (μM)^*a*^		
	SNAP		SIN-1 for promastigotes
	Promastigotes	Amastigotes	
Sb^s^ WT	117 ± 14	111 ± 22	1,242 ± 62
Sb^s^ PMM^r^ clone 8	84 ± 16	285 ± 38*	1,537 ± 31
Sb^i^ WT	239 ± 26	97 ± 5	961 ± 43
Sb^i^ PMM^r^ clone 8	96 ± 8	2,456 ± 342*	306 ± 29*
Sb^r^ WT	57 ± 8	934 ± 41	313 ± 29
Sb^r^ PMM^r^ clone 9	45 ± 1*	1,283 ± 132*	343 ± 37

a The mean IC_50_ values are from 3 separate experiments. *, *P* < 0.05, compared to the respective WT.

### Genetic variants discriminate PMM^s^ lines from PMM^r^ lines.

**FIG 5:**
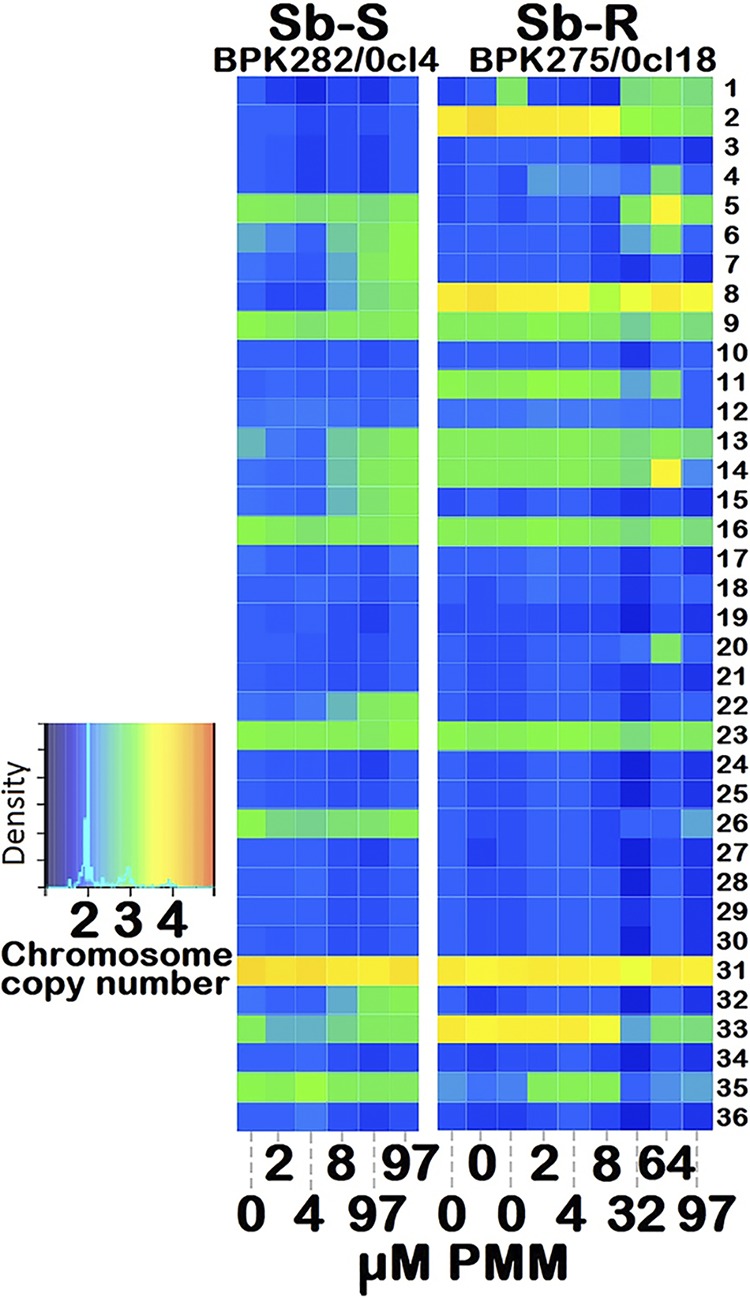
Different aneuploidy stress responses during PMM selection of the Sb^s^ (BPK282/0cl4) and Sb^r^ (BPK275/0cl18) lines. The heat map shows the copy number status of the 36 chromosomes with from 0 (wild type) to 2, 4, 8, 32, 64, and 97 μM PMM as disomic (blue), trisomic (green), or tetrasomic (ivory). The color key shows the normalized chromosome read depth and the distribution frequency. Chromosomes 6, 7, 8, 13, 14, 15, 22, and 32 (all at 8+ μM) shifted from disomy to trisomy in Sb^s^ but not Sb^r^ strains, which showed an increased somy for chromosomes 1 and 5 (at 32+ μM), but lower somy for chromosomes 2, 11, and 33 at 32+ μM and 14 at 64+ μM.

Only the Sb^s^ and Sb^r^ strains were used in genotyping studies to determine what influence the two extreme phenotypes of Sb susceptibility had on PMM^r^. We screened genome-wide data for mutations associated with PMM tolerance in the WT and PMM^r^ stages with 2, 4, 8, and 97 μM PMM for both the Sb^s^ and Sb^r^ lines, along with 32 and 64 μM PMM for the Sb^r^ line. The Sb^s^ sample had a dose-dependent aneuploidy signature: the copy number of chromosomes 6, 7, 8, 13, 14, 15, 22, and 32 (eight in total) increased from disomy in the WT to trisomy in both Sb^s^ PMM^r^ libraries at 8+ μM PMM (Fig. 5). The Sb^r^ chromosome copy numbers fell to disomy from trisomy for chromosomes 11 and 14 (at 32 μM+ and 64+ μM PMM, respectively), as well as from tetrasomy to trisomy for chromosomes 2 and 33 (at 32+ μM PMM) but rose from disomy to trisomy for chromosomes 1 and 5 (at 32+ μM PMM). Consequently, there was a marked difference in the somy-level response for the Sb^s^ and Sb^r^ isolates, highlighting that their adaptation to this drug may differ.

Transient amplification of the rRNA locus at both kb 1046 to 1053 and kb 1058 to 1065 on chromosome 27 was discovered in both Sb^s^ and Sb^r^ isolates at 2, 4, and 8 μM PMM, but this was absent in the WT line and the lines resistant to 32, 64, and 97 μM PMM (Text S2; Fig. S2 and S3). These amplified regions spanned three (LdBPK_270030120-140) and four (LdBPK_270030190-220) genes, respectively, where the first gene of each amplification was the 18S SSU rRNA gene. This increased the local rRNA gene dosage from 6 (WT strains) to 10 (strains resistant at 2 to 8 μM PMM), but this reverted back to 6 in the strains resistant at 32 to 97 μM PMM.

The remaining other genetic changes were a deletion and a heterozygous single nucleotide polymorphism (SNP). The sole deletion observed was in Sb^s^ PMM^r^ isolates and was a heterozygous loss of a 1,532-bp region containing an NAD-dependent epimerase/dehydratase family gene. The sole consistent SNP was a heterozygous one (G > A) at a mitogen-activated protein kinase kinase kinase (MAPKKK) gene (LdBPK_190007100) at chromosome 19 position 45,638 in the Sb^s^ isolate resistant to PMM at a low dose (0, 2, 4, 8 μM PMM) that became fixed in both Sb^s^ PMM^r^ isolate clones (97 μM). No SNPs were found in the Sb^r^ lines during PMM exposure. No other PMM-related SNPs, insertions, or episomes that distinguished all PMM^s^ lines from all PMM^r^ lines in either the Sb^s^ or the Sb^r^ isolates were observed.

## DISCUSSION

The results indicated that the induction of PMM^r^ parasites from a single cloned WT can result in a mixed population of parasites, based on the response of individual Sb^s^ PMM^r^ subclones and the parental drug-resistant promastigote parasites to SIN-1 or SNAP treatment. The PMM IC_50_ values for Nepalese L. donovani WT strains at the start of this study (for the Sb^s^ PMM^r^ strain, 354 ± 4 μM; for the Sb^i^ PMM^r^ strain, 56 ± 22 μM; for the Sb^r^ PMM^r^ strain, 65 ± 2 μM) were higher than those quoted for L. aethiopica clinical isolates from patients with localized cutaneous leishmaniasis (4.44 μg/ml or 7.22 μM), mucosal cutaneous leishmaniasis (21.8 μg/ml or 35.45 μM), or diffuse cutaneous leishmaniasis (0.20 μg/ml or 0.33 μM) (21).

One of the most striking phenotypes associated with PMM^r^ parasites compared to their WT strains was the increased resistance to NO, which was expressed primarily at the intracellular amastigote stage, and the Sb^s^ PMM^r^ clone had a lower resistance than the Sb^i^ PMM^r^ and Sb^r^ PMM^r^ parasites. The higher resistance to NO probably reflects adaptations related to Sb^r^, which also gives increased resistance to NO (22). A relationship between PMM^r^ and increased resistance to nitrosative stress has been reported in L. infantum at the intracellular amastigote stage (11), and an Indian L. donovani PMM^r^ clinical isolate (BHU573) that had PMM^r^ induced using the same protocol showed increased resistance to SNAP treatment at the promastigote stage but a significantly lower tolerance at the intracellular amastigote stage (23). It is possible that the clinical use of PMM as an antibiotic treatment rather than as a first-line antileishmanial treatment may have favored the survival of naturally occurring NO-resistant parasites. There is a precedent for this type of phenomenon, as genotyping of historical bacterial strains indicates that methicillin resistance was a result of using methicillin to circumvent penicillin resistance in the 1940s. The limited use of methicillin then provided the selective pressure to drive the nosocomial spread of a variant that was present in the bacterial population (24). A natural isolate of L. chagasi had a SNAP IC_50_ value of 48.8 mM, which is approximately 100 times higher than the IC_50_ obtained in this study, but the PMM sensitivity of this strain was not determined (25). There are data to indicate that selection at the amastigote stage can have a more profound effect on PMM susceptibility in L. donovani. In this study, adaptation to PMM^r^ to 97 μM PMM took 26 weeks to develop when using the promastigote stage, and studies using the AG83 L. donovani strain found that it took 3 months to select PMM^r^ using a 10 μM stepwise exposure of promastigotes to PMM up to a maximum of 200 μM. In contrast, Hendrickx et al. (10), who used the same Sb^r^ WT parent used in the present study (BPK275/0cl18), found that resistance to PMM could be induced within 2 selection cycles if the intracellular amastigotes were exposed to PMM (mean ± standard deviation [SD] IC_50_, 199.0 ± 8.5 μM). This may indicate that the selection of PMM^r^ can occur more rapidly if drug selection occurs at the amastigote stage. Therefore, it would be prudent to monitor the resistance of clinical strains to all clinically used antileishmanial drugs to screen for the evolution of multiple-drug-resistant strains.

The metabolic and lipidomic changes in the three PMM^r^ types of parasites produced in this study were different and strain specific, suggesting that PMM^r^ was mediated by different mechanisms in strains with different genetic backgrounds. There was no clear marker for the PMM^r^ from an untargeted analysis of metabolites present or a targeted analysis determining the metabolites associated with resistance to oxidative stress in *Leishmania*, i.e., glutathione and proline. Surprisingly fewer metabolic/lipidomic changes occurred for PMM^r^ strains than for MIL^r^ strains in which resistance was induced using the same parental clones (19). This may be related to the mode of action or these drugs, indicating that PMM has fewer targets in the parasite than MIL. This finding is similar to the results of Berg et al. (17) using an L. donovani strain isolated in Ethiopia (MHOM/ET/67/HU3), where PMM^r^ resulted in 14 metabolic changes compared to the metabolism of the WT, whereas MIL^r^ induced 20 changes. A recent study found that PMM^r^ was associated with increased glycolytic activity to form products that can scavenge hydrogen peroxide (26). However, the results from this study did not show any major changes in TCA cycle metabolites. PMM^r^ was associated with changes in 2-hydroxy acid production from certain amino acids. In *Leishmania*, the transamination of amino acids to their corresponding 2-keto acids involves a broad-specificity aminotransferase that can use aromatic amino acids and a branched-chain amino acid, aminotransferase (BCAT), with a specificity for leucine, isoleucine, and valine (27). The 2-hydroxy-acid dehydrogenase(s) responsible for the reduction of the 2-keto acids to form lactates has not been identified. In lactic acid bacteria (28) and protozoa, such as Trichomonas vaginalis (29), these reactions are catalyzed by the canonical NAD-dependent hydroxy acid dehydrogenase lactate dehydrogenase (LDH), an enzyme with broad substrate specificity (30). *Leishmania* species do not encode LDH, but genes for malate dehydrogenases (MDH) and a putative d-lactate dehydrogenase (d-LDH) are present. Recently, the upregulation of d-LDH and BCAT mRNA was linked to PMM^r^ in L. donovani by transcriptome sequencing (RNA-seq) analysis of WT and PMM^r^ strains (15). Overexpression of the putative d-LDH in L. donovani, L. major, and L. infantum also increased PMM resistance, and there was cross-resistance with other aminoglycosidic antibiotics. Similar results were observed following the overexpression of BCAT, although there was no additive effect when BCAT and d-LDH were coexpressed in the same line. The authors proposed that the enzymatic modification of amino or hydroxyl groups on the antibiotics could contribute to the resistance mechanisms of BCAT and d-LDH (15). In the present study, the levels of 2-keto-3-methylvaleric acid (KMVA), the 2-keto acid derivative of isoleucine, indicated increased BCAT activity in the Sb^i^ PMM^r^ strain compared to that in the corresponding WT. The high levels of hydroxyphenyl lactic acid and arginic acid in the PMM^r^ strains derived from Sb^s^ and Sb^r^ strains must result from increased 2-hydroxy acid dehydrogenase activity. These new metabolomic data support a role for increased BCAT and/or d-LDH activity in PMM^r^ in L. donovani, based on RNA-seq analysis.

Aneuploidy is hypothesized to represent an early adaptive response to drugs, but this assertion is complicated by its intrinsic dynamic nature within and across *Leishmania* species and life cycle stages to modulate the expression of the majority of genes (31, 32). Here, the higher copy number of seven chromosomes for the Sb^s^ isolates associated with 8+ μM PMM contrasted with the jump in somy for different chromosomes in the Sb^r^ isolates (chromosomes 1 and 5), along with the fall in somy for chromosomes 2, 11, and 33 at 32+ μM PMM and also for chromosome 14 at 64+ μM. This result shows again that the genetic background of the strains used in the selection experiment may influence the outcome of the consecutive molecular adaptations. This genetic diversity may also explain why PMM selection resulted in the emergence of the PMM^r^ parent and subclone with PMM IC_50_ values different from those for the Sb^s^ WT. A study comparing the efficacy of dosing L. donovani-infected patients in Sudan with PMM at 15 mg/kg of body weight for 28 days with that of dosing with PMM at 20 mg/kg for 21 days showed that peak PMM plasma levels occurred approximately 1 to 2 h after dosing and that both dosing regimens gave drug levels that were much lower than 13 μg/ml (mean values on day 1, 7.8 ± 4.9 μg/ml [i.e., 12.6 μM] with dosing at 20 mg/kg and 5.6 ± 4.2 μg/ml [i.e., 9.1 μM] with dosing at 15 mg/kg). At 6 months of follow-up, both of these regimens were associated with an 80 to 81% cure rate, indicating that some patients still harbored parasites (33). Based on our data, the remaining parasites would have been exposed to sufficient drug (i.e., >8 μM) to induce the change in gene somy required for PMM^r^.

PMM is an aminoglycoside affecting ribosome function and membrane fluidity (23), and studies have shown that PMM targets the decoding A site of the ribosome SSU, with mutations at positions 1408 and 1491 determining sensitivity to PMM (13). In this study, we found a transient rRNA locus amplification at 2 to 8 μM PMM in both Sb^s^ and Sb^r^ strains, indicating that at low doses, the 18S SSU rRNA gene transcription rate probably increases, highlighting an alternative PMM tolerance mechanism. The only consistent SNP in PMM^r^ parasites was a heterozygous one (G > A) at an MAPKKK gene in the Sb^s^ line. MAPKKK proteins activate mitogen-activated protein kinase kinase proteins through the phosphorylation of serine and serine/threonine residues in the T loop. A study in Larix olgensis found that MAPKKKs were induced by NO at the transcriptional level, and it is known that NO upregulates mitogen-activated protein kinases (34). So, perhaps it is no coincidence that in this study PMM^r^ in L. donovani was associated with increased resistance to NO induced by SNAP treatment. This is important to ascertain, as it may be possible to produce an inhibitor or provide a supplement that could reverse PMM resistance and extend the clinical life of the drug against L. donovani. In a previous study based on Cos-Seq, the copy number of the LinJ.06.1010 gene, with its leucine-rich repeat domain, was found to be responsible for the resistance to PMM (14), but this was not verified by Rastrojo et al. (15), while in the present study, we did not find a local copy number variation but did find an increase in the copy number of chromosome 6. These different results obtained with different strains might again highlight the influence of the genetic background on the outcome of molecular adaptations developed during resistance selection. Last but not least, our work is consistent with the lack of PMM-MIL cross-resistance (35), underlining the potential use of this pair as a combination treatment.

In summary, this study showed that resistance to PMM is present in an L. donovani isolate from Nepal in the absence of PMM drug pressure. It was relatively easy to induce resistance in L. donovani, and the subclones produced from the parental PMM^r^ line could express different levels of PMM resistance. PMM^r^ was associated with enhanced resistance to NO, the mechanisms that control PMM^r^ varied between lines, and the Sb resistance profile of the parasite, together with its genetic background, had an impact on the outcome of selection. The PMM^r^ strains had significantly higher levels of either the keto acid KMVA or 2-hydroxy acids (arginic acid and HPLA), which may indicate the upregulation of BCAT or d-lactate dehydrogenase, activities recently linked to PMM^r^. The challenge now is to expand this study to a larger number of field isolates to determine the predominant phenotype present in areas of endemicity.

## MATERIALS AND METHODS

### Reagents.

Pure crystalline PMM (paromomycin sulfate USP) was supplied from Gland Pharma, India. Resazurin, SNAP, Giemsa stain, and lipopolysaccharide were purchased from Sigma-Aldrich (Gillingham, UK). HOMEM medium, RPMI 1640, phosphate-buffered saline (PBS), pH 7.4, penicillin-streptomycin, glycine, and fetal calf serum were obtained from Invitrogen, Paisley, UK. SDM medium was custom made by Gibco, Paisley, UK. *S*-Nitroso-*N*-acetyl-d,l-penicillamine (SNAP) and 3-morpholinosydnonimine (SIN-1) were purchased from Enzo Life Sciences, Exeter, UK. All other reagents were analytical grade.

### Animals and parasites.

Age-matched inbred BALB/c female mice (weight, 20 to 25 g) were used in studies at Strathclyde University. Animal studies were carried out with local ethical approval and had UK Home Office approval. L. donovani cloned strains with different Sb susceptibility backgrounds were derived from isolates obtained from VL patients at the B. P. Koirala Institute of Health Sciences, Dharan, Nepal: MHOM/NP/02/BPK282/0cl4 (Sb^s^). MHOM/NP/02/BPK087/0cl11 (Sb intermediate [Sb^i^]), and MHOM/NP/02/BPK275/0cl18 (Sb^r^) (16).

### Selection of PMM^r^ clones.

L. donovani strains were adapted to grow in a stepwise manner in increasing concentrations of PMM (2, 4, 8, 16, 32, 64, 97 μM) until all lines grew at rates similar to the rate for the wild-type (WT) parasites in HOMEM medium supplemented with 20% (vol/vol) fetal calf serum (complete HOMEM). The highest concentration of PMM used was 97 μM, as the parasites did not grow well in the presence of higher drug concentrations. Parasites selected to grow as well as the WT in 97 μM PMM were termed PMM resistant (PMM^r^), and their specific designation depends on the WT used; i.e., the Sb^s^ PMM^r^ parental line was derived from BPK282/0cl4, the Sb^i^ PMM^r^ parental line was derived from BPK087/0cl11, and the Sb^r^ PMM^r^ parental line was derived from BPK275/0cl18. Clones from the parental drug-resistant line were isolated, expanded in Locke medium on top of Tobie’s agar base, and then grown in complete HOMEM. A stock solution of aqueous PMM (3 mg/ml, freshly prepared every 3 months) was stored at −20°C and used to prepare drug selection medium. Clones were produced from the parental PMM^r^ lines using the microdrop technique (10) and designated with a number.

### Genetic characterization of Sb^s^ PMM^r^ and Sb^r^ PMM^r^ strains.

Cell harvesting, lysis, genomic DNA isolation, and genome sequencing were completed as outlined previously (19). Genomic DNA was sequenced on an Illumina HiSeq 2000 platform, with the median sequence coverage being 47.4 ± 17.8 reads per site averaged across 15 sequence libraries (see Table S2 in the supplemental material posted at https://doi.org/10.15129/88f47c26-ff93-4a7c-8c17-d323aa24052e). Following screening for contamination, the DNA reads for each library with insert sizes of less than 1,000 bases were mapped to a new L. donovani LdBPK282 reference genome using the Smalt program with exhaustive alignments (v0.7.4; https://sourceforge.net/projects/smalt/) (35). All nonmapping and PCR duplicate reads were removed. Sites with low-quality scores, in repetitive regions, with poor mapping scores, in low-complexity regions, with significant forward-reverse strand amplification bias, or with low read coverage were excluded. Chromosome copy number variation and scans for large copy number variations and episomes were performed based on the per cell read depth, to reflect gene dosage, as outlined previously (36). Changes in chromosome copy numbers were detected by comparing the distributions of coverage levels across all sites for each chromosome using *t* tests, as outlined elsewhere (37). SNPs, small insertions, and deletions were called using the population-based Unified Genotyper method in the Genome Analysis Toolkit (v3.4) with the parameters described previously (37).

### Phenotyping of promastigotes.

The effects of amphotericin B (AMB), PMM, SIN-1, and SNAP on the growth of the promastigotes were determined using a colorimetry-based assay according to the method described by Shaw et al. (19). Parasites (5 × 10^5^ parasites/100 μl, *n* = 6/treatment) were exposed at time zero to AMB (1 μM) or various concentrations of PMM, SIN-1, or SNAP (*n* = 6/treatment) for 1 h (SIN-1 studies) or 72 h (AMB, PMM, or SNAP studies) at 27°C. After 1 h of exposure, the SIN-1 solution was removed, fresh medium was added, and the cells were incubated for a further 71 h. In some studies, proline (200 mM) was added to the medium with or without PMM solution, as this proline concentration was used in previous studies investigating the role of proline in drug resistance in L. donovani (17). Parasites were exposed to AMB to show whether resistance to PMM was drug specific. Resazurin solution (20 μl/sample, 0.0125% [wt/vol] PBS, pH 7.4) was added to the samples for the last 18 h of culture, and then the absorbance of samples at 550 to 590 nm was determined using Softmax Pro (v2.0) software. The effect of drug treatment on cell viability was determined by calculating the mean suppression compared to the relevant mean control value, and this was used to determine the IC_50_ for a particular drug/compound using Grafit (v5) software.

### Metabolic and lipidomic characterization studies.

Extraction of the lipids or metabolites was carried out using 4 × 10^7^ L. donovani promastigotes/strain obtained on day 4 of culture using the methods described by Shaw et al. (19). Specific metabolites were identified using IDEOM, and 4 standard mixes were used in assays, whereas lipids were identified using Xcalibur software (v2.2) and an in-house Excel macro. PCA and OPLS-DA plots were produced using published methods (38) and SIMCA software (v14.1). The experiments were repeated three times, but data from one single representative experiment performed using all samples in one run are shown.

### Phenotyping of intracellular amastigotes.

The method for the phenotyping of intracellular amastigotes described by Carter et al. (22) was followed. Briefly, peritoneal macrophages (0.5 × 10^5^/sample, *n* = 5/treatment) from BALB/c female mice were infected with L. donovani promastigotes at a host/parasite ratio of 20:1 for 24 h. Infected cells were incubated for 72 h with medium alone (controls), SNAP (10 to 160 μg/ml), or IFN-γ and LPS (IFN-γ, 50 or 100 U/ml; LPS, 50 or 100 ng/ml). The percentage of infected macrophages and the mean number of parasites per host cell were determined from 200 randomly selected infected cells; infectivity was determined by multiplying both parameters. The effect of treatment on parasite survival was determined as the mean percent suppression compared to the relevant mean control value. Experiments were repeated a minimum of three times.

### Statistical analysis of data.

The effect of drug treatment on cell proliferation in *in vitro* drug studies was analyzed using a Mann-Whitney U test for comparing two treatments or a Kruskal-Wallis test followed by Dunn’s *ad hoc* test for determination of statistically significant differences between three or more treatments (Statview [v5.0.1] software package). Results were considered significant at a *P* value of <0.05.

### Data availability.

Supplemental data and data underpinning this publication are available from the University of Strathclyde KnowledgeBase at https://doi.org/10.15129/88f47c26-ff93-4a7c-8c17-d323aa24052e. Raw sequence reads are available from the European Nucleotide Archive (ENA) via accession number ERP115194. All data used in this study have been deposited under BioProject accession number PRJEB32497 as Sequence Read Archive accession numbers ERS197378, ERS161532, ERS160178, ERS160177, ERS160176, ERS197372, ERS340112, ERS340111, ERS340110, ERS197378, ERS161532, ERS160178, ERS160177, ERS160176, and ERS197372 (see also Table S4 posted at https://doi.org/10.15129/88f47c26-ff93-4a7c-8c17-d323aa24052e).
